# Immersive experience scene quality and Environmental Resilience Support in digitally mediated immersive tourism settings: the roles of ecological presence, awe, psychological ownership, and self-efficacy

**DOI:** 10.3389/fpsyg.2026.1910332

**Published:** 2026-10-08

**Authors:** Guanxi Chen, Jiajun Chen

**Affiliations:** 1 School of Design, NingboTech University, Ningbo, China; 2 Department of Business Administration, Joongbu University, Goyang, Republic of Korea; 3 School of Business, Nantong Institute of Technology, Nantong, China

**Keywords:** digitally mediated tourism, ecological awe, ecological presence, Environmental Psychological Ownership, Environmental Self-Efficacy, immersive experience quality, resilience-support intentions

## Abstract

Digitally mediated immersive tourism environments increasingly integrate technologies such as VR, interactive media, digital-human applications, and, in some cases, GenAI-related functions. Visitors typically encounter these technologies as part of a broader scene rather than as isolated features. This study therefore focuses on Immersive Experience Scene Quality (IESQ) in digitally mediated immersive tourism settings and examines its association with Environmental Resilience Support (ERS). Drawing on environmental psychology and a responsibility–efficacy framework informed by the Norm Activation Model and the Theory of Planned Behavior, the study conceptualizes Ecological Presence (EP) and Ecological Awe (EA) as parallel psychological gateways linking scene quality to Environmental Psychological Ownership (EPO), Environmental Self-Efficacy (ESE), and ERS. Survey data were collected from 520 visitors at two immersive tourism settings in China and analyzed using PLS-SEM, artificial neural networks, and necessary condition analysis, with follow-up interviews used for contextual interpretation. The results show that narrative coherence and system usability are consistently associated with both EP and EA, while multisensory integration is more closely related to EP and perceived realism to EA. Interactive Participatory Affordance is not significantly associated with either gateway, suggesting that interaction may depend on how closely it is integrated with ecological meaning. EP and EA are both associated with EPO and ESE, which in turn are positively associated with ERS. The findings further indicate that responsibility and perceived action capability are important correlates of resilience-oriented support, while no single antecedent constitutes a practically meaningful necessary condition. Overall, the study shifts attention from technological novelty to the organization of ecological meaning, situated experience, responsibility, and action capability, and extends immersive tourism research toward governance-oriented environmental outcomes.

## Introduction

Immersive tourism settings increasingly combine physical place, digital mediation, narrative interpretation, and visitor participation. These settings are not only entertainment spaces; they may also influence how visitors notice ecological cues, interpret environmental risks, and evaluate their own role in environmental protection and resilience governance. Environmental resilience refers to the capacity of destination environmental systems to maintain essential functions, withstand shocks, and recover or adapt under tourism-related disturbances and external risks (Wani et al., 2024). From an environmental psychology perspective, public support for such resilience may be associated with whether visitors feel situated within ecological conditions, experience concern or awe, regard environmental issues as personally relevant, and believe that individual or collective action can make a difference. Immersive tourism experiences may therefore provide an important psychological context for resilience-oriented public support (Liu et al., 2024).

Tourism experiences are also being reshaped by immersive and digitalized environments. Multisensory presentation, interactive participation, and narrative guidance have shifted visitor experience from passive viewing and information acquisition toward situated involvement, meaning construction, and emotional engagement (Pencarelli, 2020). More recently, some digitally mediated settings have also incorporated GenAI-related functions such as adaptive narrative generation, conversational interpretation, personalized content, and real-time information provision (Nicolau, 2025; Ilieva et al., 2024; Sánchez-Martín et al., 2025). In practice, visitors encounter digital and AI-enabled functions as part of a broader scene rather than as isolated technologies, and their evaluations are likely to center on whether narratives are coherent, ecological cues are credible, interaction is meaningful, and system operation is continuous. The present study therefore examines perceived immersive scene quality within digitally mediated immersive tourism settings and its associations with ecological psychological responses and Environmental Resilience Support.

Existing research has connected immersive technologies, particularly virtual-reality tourism and virtual nature experiences, with pro-environmental attitudes and behavioral intentions, often positioning presence as an important explanatory construct. Recent studies have further developed the concept of ecological presence, referring to the embodied sense of being situated with natural and ecological elements in immersive environments, and have linked it to environmentally responsible outcomes (Su et al., 2024). Nevertheless, three gaps remain. First, existing studies tend to focus on general pro-environmental intention rather than support for resilience-oriented governance involving resistance, recovery, adaptation, restoration, and management measures. Second, ecological presence is frequently treated as the principal psychological pathway, while the distinct affective role of ecological awe remains insufficiently integrated. Third, the indirect associations linking immersive experience and ecological emotion with governance-oriented support remain undertheorized, particularly with regard to Environmental Psychological Ownership and Environmental Self-Efficacy.

Drawing on environmental psychology and sustainable behavior research, two psychological pathways are especially relevant. The first is a self-transcendent emotional pathway. Immersive ecological experiences may be associated with ecological awe, an affective response to the perceived vastness, fragility, and order of natural systems. Awe may shift attention away from narrowly self-focused concerns and toward broader ecological systems and collective interests, thereby strengthening care for and stewardship of nature (Xu and Hu, 2024). The second is a responsibility- and capability-related pathway. When individuals experience a sense of psychological connection and responsibility toward the environment—conceptualized as Environmental Psychological Ownership—they may also report stronger confidence in their capacity to support environmental action, namely Environmental Self-Efficacy (Xu et al., 2023). Together, these constructs offer a theoretically ordered explanation of how immersive ecological experiences may be associated not only with temporary emotional responses, but also with more stable support orientations.

Following this logic, the present study conceptualizes Ecological Presence and ecological awe as two parallel psychological gateways. Ecological Presence reflects situated entry into ecological cues and sustained attention to environmental information, whereas ecological awe captures a self-transcendent emotional response to the vastness, fragility, and order of natural systems. Both are linked to Environmental Psychological Ownership and Environmental Self-Efficacy, which in turn are associated with Environmental Resilience Support. Because all constructs were measured at a single point in time, the proposed ordering is used as a theory-guided association structure rather than as a temporal sequence.

To examine these relationships in a measurable and design-relevant way, this study treats Immersive Experience Scene Quality (IESQ) as the principal antecedent and decomposes it into five dimensions: Multisensory Integration Quality, narrative guidance and coherence, Interactive Participatory Affordance, perceived realism cues, and system usability. This approach conceptualizes immersion as a structured field experience composed of specific design and operational features. In digitally mediated immersive tourism settings, these dimensions capture visitors’ evaluations of overall scene organization and experience delivery; they are not measures of any specific technology, including GenAI capability itself.

This study makes three contributions. First, it extends immersive experience research beyond satisfaction, revisit intention, and general pro-environmental intention by focusing on Environmental Resilience Support, a governance-oriented outcome involving support for protection rules, restoration measures, long-term investment, public-resource allocation, and adaptive management. Second, it develops a dual-gateway framework that links Ecological Presence and ecological awe with resilience support through Environmental Psychological Ownership and Environmental Self-Efficacy. Rather than reproducing the complete NAM and TPB structures, the study selectively connects the responsibility logic associated with NAM and the perceived-control logic associated with TPB. Third, it specifies a five-dimensional structure of immersive scene quality and examines this structure across two digitally mediated immersive tourism settings, allowing the study to compare which experiential features are more consistently associated with ecological psychological responses and whether interaction is beneficial only under particular design conditions.

The remainder of the paper proceeds as follows. The next section develops the theoretical background and hypotheses. The study then presents the research design, analytical procedures, and empirical results. The final sections discuss the theoretical and managerial implications, acknowledge the limitations of the research design, and identify directions for future research.

## Literature review and hypothesis

### Theoretical foundation: a responsibility–efficacy framework informed by NAM and TPB

This study conceptualizes Environmental Resilience Support (ERS) as a governance-oriented public support orientation rather than as general environmental friendliness, satisfaction, or revisit intention. ERS involves individuals’ readiness to endorse, cooperate with, and sustain policies and practices that enhance the resistance, recovery, and adaptation capacity of destination ecosystems. To explain this outcome, the framework draws selectively on two complementary psychological logics: responsibility internalization associated with the Norm Activation Model (NAM) and perceived action capability associated with the Theory of Planned Behavior (TPB). These two logics provide a focused basis for explaining how environmental issues become personally consequential and how individuals judge their capacity to respond.

Norm Activation Model contributes the responsibility-oriented component of the framework. Environmental support becomes more likely when ecological consequences are recognized as personally relevant and responsibility is internalized, because awareness of environmental consequences and ascribed responsibility can strengthen willingness to adopt pro-environmental actions (Ribeiro et al., 2025). Here, this process is represented by Environmental Psychological Ownership (EPO), which captures the extent to which environmental conditions are perceived as connected to the self, worthy of concern, and requiring protection. Psychological ownership of natural places has likewise been associated with perceived responsibility and conservation behavior (Wang et al., 2023). The analysis focuses on this proximal self-relevance and responsibility process rather than separately modeling the full NAM sequence of awareness of consequences, ascription of responsibility, and personal norm (Steg and de Groot, 2010).

Theory of Planned Behavior contributes a complementary action-feasibility logic. Recognition of environmental responsibility may be insufficient when individuals doubt whether meaningful action is possible. This capability-related process is represented by Environmental Self-Efficacy (ESE), referring to confidence in one’s ability to overcome barriers and undertake effective environmental action (Plohl et al., 2026). ESE is related to perceived behavioral control but focuses specifically on perceived capability, rather than the broader combination of internal capability and external constraints. Together, EPO and ESE form a responsibility–efficacy structure: EPO reflects the belief that an environmental issue is personally relevant and deserves responsibility, whereas ESE reflects confidence in being able to contribute effectively. Immersive Experience Scene Quality and ecological value orientations are positioned as antecedent conditions associated with these beliefs through ecological presence and ecological awe. The proposed EPO–ESE linkage captures a transition from responsibility-related self-relevance to perceived action capability: when environmental conditions become personally consequential, individuals may be more motivated to understand feasible responses and sustain engagement (Yang et al., 2025). Attitude, subjective norm, behavioral intention, and the broader perceived behavioral control construct are therefore outside the present framework, whose purpose is to examine this more focused responsibility–efficacy process.

### Immersive experience scene quality in digitally mediated immersive tourism settings: operational boundary

A recurring issue in immersive-experience research is whether immersion should be treated as an experiential outcome or as a set of designable input conditions. When the focus is on sustainability-oriented psychological mechanisms and behavioral support, a more appropriate approach is to treat immersive experience as a psychological process triggered by scene elements and to conceptualize scene quality itself as the antecedent that can be designed and managed. This distinction is consistent with immersive media research, which generally views outcomes such as presence, engagement, emotion, and attitudes as driven by upstream conditions including sensory fidelity, interaction structure, and narrative organization (Han et al., 2024; Kieanwatana and Vongvit, 2024).

This distinction is especially relevant in digitally mediated tourism settings, where GenAI-related functions may operate alongside other immersive technologies. Adaptive narrative generation, personalized interpretation, conversational guidance, and real-time content provision can contribute to the organization and delivery of immersive experiences (Florido-Benítez, 2024; Tortora et al., 2025; Sánchez-Martín et al., 2025). In the present framework, GenAI-related functionality is treated as one possible element of a broader digital technological context, while the empirical focus remains on visitors’ perceptions of scene organization and experience delivery. The five IESQ dimensions therefore operationalize perceived scene quality rather than technology-specific capability or adoption intensity.

The field design captures naturally occurring visitor experiences in these settings. Individual exposure to particular digital and GenAI-related functions was not standardized, and the analysis does not compare specific technological configurations or isolate the contribution of GenAI. The results are therefore interpreted at the level of immersive scene quality within digitally mediated immersive contexts, which is the level at which visitors encountered and evaluated the experience.

Based on this rationale, the independent variable is defined as Immersive Experience Scene Quality (IESQ) and operationalized as a five-dimensional input structure. This approach is consistent with experience-quality research in virtual exhibitions and digital heritage, where front-end experience quality is commonly decomposed into multiple designable components rather than treated as a single undifferentiated construct (Pratisto et al., 2023). In the present study, five dimensions are specified: Multisensory Integration Quality (MIQ), Narrative Guidance and Coherence (NGC), Interactive Participatory Affordance (IPA), Perceived Realism Cues (PRC), and System Usability/Seamlessness (SU). Together, these dimensions capture tourists’ integrated evaluations of sensory coordination, meaning guidance, interaction opportunity, situational credibility, and technological continuity during immersion (Melo et al., 2022; Meng and Dolah, 2025; Nguyen et al., 2023; Choi and Nam, 2024; Nguyen, 2025).

Multisensory Integration Quality refers to the consistency and coordination of visual, auditory, and spatial or haptic cues, which provide the perceptual basis for “being there” and reduce awareness of mediation (Melo et al., 2022). NGC concerns the structured organization and temporal continuity of interpretive content; clearer and more coherent narratives facilitate ecological meaning-making and help visitors integrate environmental information into causal understanding and value judgment (Meng and Dolah, 2025). IPA captures whether visitors can meaningfully engage with the experience through clear entry points, timely feedback, and manageable participation thresholds, thereby shaping agency and involvement (Nguyen et al., 2023). PRC refers to credible and internally consistent cues that make the experience world psychologically acceptable as a believable situation, thereby enhancing perceived authenticity, credibility, and meaningfulness (Choi and Nam, 2024). Finally, SU reflects technological accessibility and continuity; even strong content and narrative design may fail to sustain immersion when systems are unstable, lagging, or difficult to use (Nguyen, 2025).

In the present model, these five dimensions of IESQ are treated as direct antecedents of Ecological Presence (EP), a presence subdimension that reflects a “being-there” experience specifically anchored in natural–ecological elements and has been linked to environmentally responsible outcomes (Su et al., 2024). Within the proposed association structure, higher perceived scene quality is expected to be associated with greater perceptibility, credibility, and continuity of ecological cues, making sustained attention to the ecological situation more likely.

Accordingly, the following hypotheses are proposed:

H1a: Multisensory Integration Quality (MIQ) is positively associated with Ecological Presence (EP).

H1b: Narrative Guidance and Coherence (NGC) is positively associated with Ecological Presence (EP).

H1c: Interactive Participatory Affordance (IPA) is positively associated with Ecological Presence (EP).

H1d: Perceived Realism Cues (PRC) are positively associated with Ecological Presence (EP).

H1e: System Usability (SU) is positively associated with Ecological Presence (EP).

### Ecological presence

In immersive media research, presence is widely regarded as a key mechanism linking technological presentation to psychological and behavioral outcomes. It refers to the subjective experience of “as if being there” within a mediated environment and functions as an upstream psychological gateway through which external cues enter deeper meaning-making processes (Su et al., 2024; Barranco Merino et al., 2023). However, conventional presence concepts mainly emphasize general spatial or media presence (Lindemann and Schünemann, 2020) and do not fully capture whether individuals psychologically enter the ecological object itself. To address this limitation, recent work has proposed Ecological Presence as an object-oriented subdimension of presence, referring to the intensity with which visitors experience themselves as being with natural–ecological elements in an immersive setting (Su et al., 2024). This concept anchors presence specifically to ecological cues and therefore provides a more direct explanation of how environmental information is attended to, trusted, and internalized.

Mechanistically, ecological presence links immersive inputs to subsequent psychological responses in three ways. First, it concentrates attention on ecological cues and reduces distraction, making environmental information more likely to enter sustained rather than superficial processing (Hicks et al., 2020). Second, it strengthens situational credibility and realism judgments, increasing the likelihood that ecological signals are treated as meaningful and worthy of serious consideration (Fauville et al., 2026). Third, it is associated with stronger involvement and role entry, shifting individuals from detached observers to situated participants and thereby providing a basis for emotional engagement, responsibility attribution, and action readiness (Hurrell et al., 2024). Ecological presence should therefore be understood not simply as experiential intensity, but as an entry mechanism through which ecological meaning enters the individual’s psychological structure.

Ecological presence is shaped not only by immersive inputs, but also by relatively stable value orientations. Nature Relatedness reflects affective and cognitive connectedness to nature; when it is stronger, ecological cues are more readily perceived as self-relevant and are more likely to become the focus of attention and interpretation (Brambilla et al., 2024). Environmental Concern similarly reflects sensitivity to ecological risks and sustainability issues. Individuals high in Environmental Concern are more likely to process immersive ecological information through a problem–consequence–responsibility frame, thereby strengthening sustained ecological processing and situational realism judgments (Meijers et al., 2023; Ibrahim et al., 2025). In this sense, ecological presence is jointly shaped by immersive conditions and value-based attentional preferences.

In the present model, ecological presence and ecological awe are conceptualized as parallel rather than sequential mechanisms. Ecological presence captures situated entry and sustained processing, whereas ecological awe reflects self-transcendent emotional activation. Although presence can facilitate awe, immersive ecological settings may also evoke awe directly through narrative, multisensory, or realism cues, and heightened awe may in turn deepen subjective immersion. A parallel specification therefore provides a parsimonious way to represent two distinct but potentially interacting psychological responses without imposing a fixed temporal order.

Accordingly, the following hypotheses are proposed:

H2a: Nature Relatedness is positively associated with Ecological Presence.

H2b: Environmental Concern is positively associated with Ecological Presence.

### Ecological awe

In sustainable tourism and environmental psychology, information provision and rational persuasion alone are often insufficient to generate stable pro-environmental support. By contrast, self-transcendent emotions are increasingly viewed as important drivers of environmental internalization and behavioral commitment. Among them, awe has received growing attention because it is typically elicited by perceived vastness and the need for accommodation, often producing a small-self perspective, a heightened sense of connection to larger systems, and a shift from self-focused concerns toward broader collective or ecological concerns (Keltner and Haidt, 2003; Song et al., 2023; Wu et al., 2024). In immersive ecological contexts, Ecological Awe refers to the awe emotion elicited by encountering the vastness, complexity, and fragility of natural ecosystems. It involves not only wonder, but also humility, respect for natural order, and a stronger orientation toward protection (Xu and Hu, 2024). Unlike general pleasure or excitement, ecological awe is more likely to orient individuals toward the ecosystem as a whole and to enhance receptivity to ecological protection and environmental governance. In the present model, ecological awe is therefore conceptualized as a parallel psychological pathway distinct from ecological presence, rather than as a downstream emotional derivative of presence. This specification does not deny the possibility that ecological presence may contribute to ecological awe; rather, it avoids treating that relationship as a necessary or temporally ordered process in the absence of longitudinal or experimental evidence.

The antecedents of ecological awe stem from both immersive scene quality and stable value traits. From the scene side, multisensory consistency, coherent narrative organization, credible realism cues, accessible interaction, and smooth system performance can jointly strengthen the perceptibility and interpretability of ecological vastness, order, and vulnerability, thereby increasing the likelihood of awe activation (Chang and Suh, 2025; Chirico et al., 2024). In settings that incorporate GenAI-related functions, adaptive storytelling and conversational explanation may contribute to more intelligible and experientially vivid ecological interpretation (Lau et al., 2025), although such functions are not directly measured in the present study. From the individual side, Nature Relatedness and Environmental Concern shape emotional susceptibility and interpretive orientation. Individuals with stronger Nature Relatedness are more likely to perceive nature as self-relevant and to experience stronger ecological connectedness (Desrochers et al., 2024), whereas those with higher Environmental Concern are more likely to interpret immersive ecological information through a risk–responsibility frame and to respond more strongly to ecological fragility and governance necessity (Kikko and Ishigaki, 2025). Thus, ecological awe is associated with both immersive cues and value-based emotional readiness.

Ecological awe is expected to be positively associated with Environmental Resilience Support through two theoretically specified indirect routes. First, by evoking a small-self perspective and a stronger sense of connectedness to ecological systems, awe may facilitate Environmental Psychological Ownership (EPO)—the sense that the environment matters to oneself and ought to be safeguarded (Peck et al., 2021). Second, ecological awe may strengthen Environmental Self-Efficacy (ESE). Self-transcendent emotions can increase moral motivation, meaning orientation, and action readiness, particularly when immersive experiences provide understandable causal structures and plausible action pathways (Fu et al., 2022). In this sense, ecological awe offers an affective route from scene quality and ecological orientations to responsibility- and capability-related beliefs, operating alongside ecological presence.

Based on the above reasoning, the following hypotheses are proposed:

H3a: Multisensory Integration Quality is positively associated with Ecological Awe.

H3b: Narrative Guidance & Coherence is positively associated with Ecological Awe.

H3c: Interactive Participatory Affordance is positively associated with Ecological Awe.

H3d: Perceived Realism Cues are positively associated with Ecological Awe.

H3e: System Usability is positively associated with Ecological Awe.

H4a: Nature Relatedness is positively associated with Ecological Awe.

H4b: Environmental Concern is positively associated with Ecological Awe.

H5: Ecological Awe is positively associated with Environmental Psychological Ownership.

H6: Ecological Awe is positively associated with Environmental Self-Efficacy.

### Environmental Psychological Ownership and Environmental Self-Efficacy

Within the present framework, Environmental Psychological Ownership (EPO) represents the point at which environmental conditions become self-relevant and responsibility-laden, consistent with the responsibility logic of NAM. Environmental Self-Efficacy (ESE), by contrast, captures confidence in one’s capacity to undertake effective environmental action, consistent with the capability-related logic associated with TPB. This distinction clarifies why responsibility recognition and action confidence are treated as complementary psychological bases of ERS. Empirical evidence similarly suggests that responsibility-related norms and self-efficacy can make distinct contributions to pro-environmental behavior, with efficacy helping translate motivation into action (Qiu, 2025).

Ecological presence is also expected to be positively associated with Environmental Psychological Ownership. When visitors experience ecological conditions as spatially immediate, perceptually vivid, and personally involving, the environment may become less distant and more closely connected to the self. This situated sense of “being there” can increase perceived self–environment connection and make ecological risks feel personally relevant, thereby strengthening concern, responsibility, and a sense of psychological ownership toward the environmental setting (Rodríguez-Ardura et al., 2025). In the present model, ESE is expected to be associated with two theoretically specified routes. First, ecological presence may strengthen self-efficacy through an understanding–control route. When individuals experience stronger ecological presence, they attend more closely to ecological cues and process immersive feedback, action prompts, and causal explanations more deeply, thereby strengthening the sense that environmental action is understandable and feasible (Sungur et al., 2025). Second, Environmental Psychological Ownership may reinforce ESE through a responsibility-based route. When the environment is internalized as a shared asset that is related to the self, individuals become more motivated to acquire relevant knowledge, explore actionable responses, and persist in engagement, which gradually strengthens the belief that they can make a meaningful difference (Chen et al., 2025). In this sense, EPO provides a more stable motivational basis for ESE, allowing efficacy beliefs to develop beyond short-lived situational confidence. This relationship therefore specifies a theoretically distinct transition from “this environmental issue concerns me and deserves my responsibility” to “I am capable of contributing effectively.” In this sense, EPO supplies the responsibility-related motivation to engage, while ESE captures the perceived capability to translate that motivation into feasible environmental action. The proposed ordering does not reproduce a NAM-to-TPB sequence; rather, it connects two narrower psychological processes that are conceptually compatible with the responsibility logic of NAM and the capability logic associated with TPB.

Accordingly, the following hypotheses are proposed:

H7: Ecological Presence is positively associated with Environmental Psychological Ownership.

H8: Ecological Presence is positively associated with Environmental Self-Efficacy.

H9: Environmental Psychological Ownership is positively associated with Environmental Self-Efficacy.

### Environmental Resilience Support (ERS)

In destination governance research, tourists’ pro-environmental orientation is often used to explain environmental attitudes or intentions. However, such indicators do not fully capture the governance challenges destinations face under environmental pressure, disruption, and uncertainty—namely, how to sustain the long-term co-existence of ecosystem functioning and tourism activities (Ahmad et al., 2020). From a resilience perspective, the key issue is not only whether damage can be avoided, but whether destination ecosystems can resist shocks, recover, and adapt while maintaining essential functions (Syed Zainal Yussof et al., 2021). In this sense, sustainability-oriented destination governance requires more than tourists’ general willingness to behave in environmentally friendly ways; it also requires their support for governance arrangements and collective actions that strengthen environmental resilience.

Accordingly, this study defines the dependent variable as Environmental Resilience Support (ERS), referring to tourists’ supportive orientation toward policies, management practices, and public actions aimed at enhancing destination ecosystem resilience. Unlike general pro-environmental behavioral intention or green consumption intention, ERS emphasizes support for resistance-, recovery-, and adaptation-oriented governance measures. In the measurement design, ERS is represented by support for resilience-enhancing policies, acceptance of long-term investment, willingness to support the use of public resources to strengthen ecosystems’ capacity to cope with and recover from shocks, and cooperation with management rules such as capacity control and protection regulations. By anchoring the outcome in these policy- and governance-related indicators, the study connects immersive experience research more directly to the practical needs of sustainable destination management.

Within the proposed framework, ERS is expected to be jointly associated with responsibility attribution and action capability. On the one hand, resilience support often requires individuals to accept constraints or costs for collective environmental benefit, making Environmental Psychological Ownership (EPO) important because it transforms environmental issues into a self-relevant responsibility and reduces psychological distance from public-goods governance (Chen et al., 2025). On the other hand, even when tourists recognize the necessity of resilience governance, support may remain unstable if they do not believe they can act effectively. Environmental Self-Efficacy (ESE) therefore serves as a critical proximal antecedent, because it strengthens perceived feasibility, participation confidence, and willingness to sustain engagement with governance-oriented action (Plohl et al., 2026; Zhang and Cao, 2025). In this sense, ERS is not a simple attitudinal outcome, but a form of governance support grounded in both responsibility commitment and capability belief.

Accordingly, the following hypotheses are proposed:

H10: Environmental Psychological Ownership is positively associated with Environmental Resilience Support.

H11: Environmental Self-Efficacy is positively associated with Environmental Resilience Support.

In sum, the model shown in Figure 1 links immersive scene quality and ecological orientations with two psychological gateways—ecological presence and ecological awe—and with the responsibility–efficacy processes represented by EPO and ESE. This focused structure provides the basis for the empirical analysis of Environmental Resilience Support.

**FIGURE 1:**
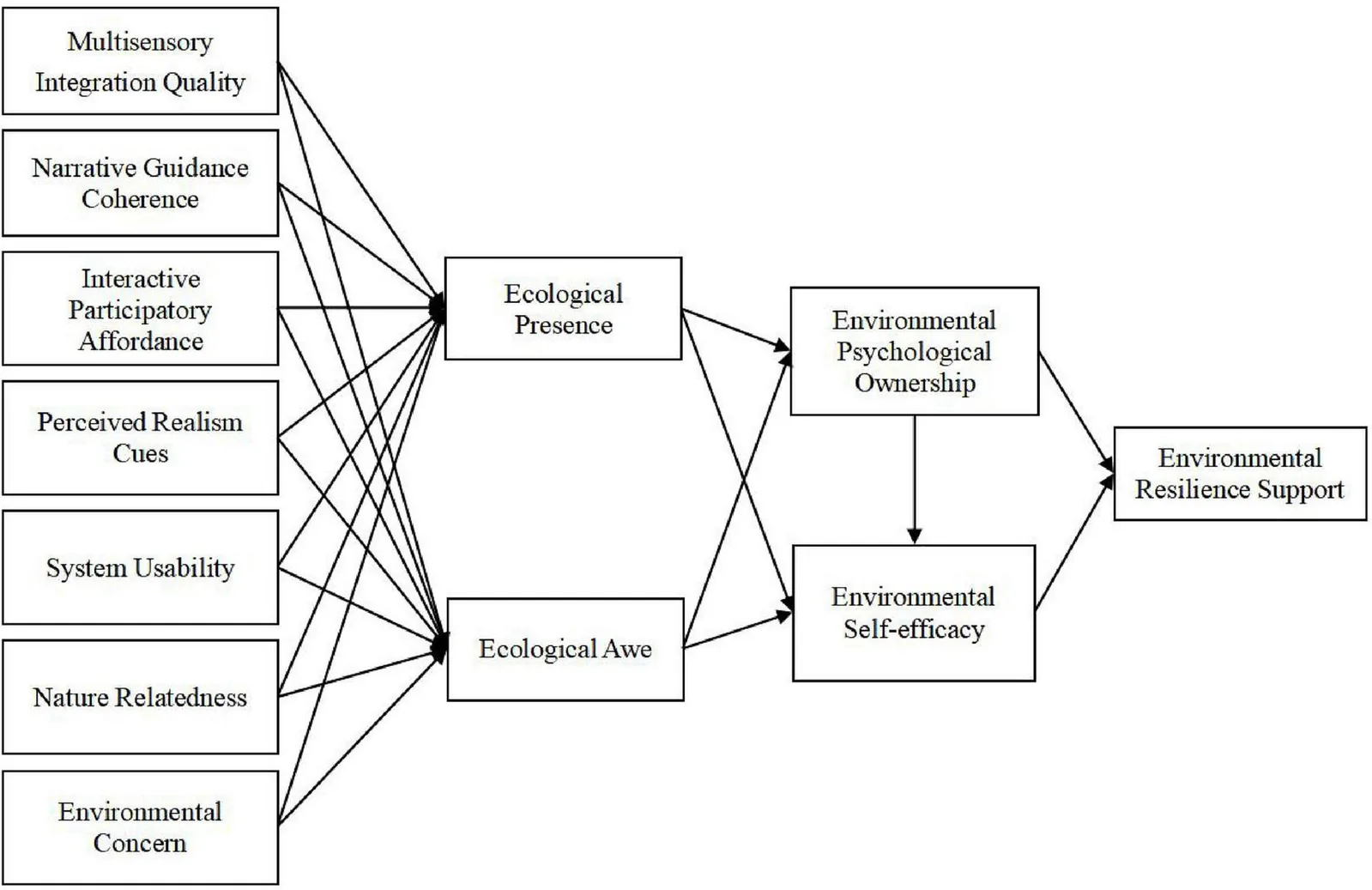
Model.

## Materials and methods

### Case

This study focused on two immersive tourism settings in China: Hangzhou M511 Light and Shadow Hub and the immersive digital consumption space in Minghe Ancient Town Scenic Area, Cixi, Ningbo. These sites were selected because they combine digitally mediated interpretation, immersive scene orchestration, and visitor participation, making them appropriate contexts for examining how immersive experience quality is translated into ecological presence, ecological awe, and resilience-support intentions.

The first site, Hangzhou M511 Light and Shadow Hub, is an indoor digital-entertainment complex centered on large-scale immersive media. Its experience portfolio includes 8K ultra-high-definition projection, location-based VR, immersive performance, and interactive digital exhibition formats. These features make sensory coordination, narrative sequencing, perceived realism, and system continuity particularly salient aspects of the visitor experience.

The second site, the immersive digital consumption space in Minghe Ancient Town, is embedded in a historic scenic-area setting and integrates digital interaction with local traditional Chinese medicine culture. Its visitor experiences include AI-enabled interactive applications, 3D digital-human interpretation, AR-based exploration, immersive light-and-shadow displays, and other interactive cultural-tourism activities. Compared with M511, the site is more spatially distributed and closely tied to heritage interpretation and destination-based visitation.

Across the two settings, digital technologies formed part of broader interpretation and content-delivery systems, but their specific configurations differed. GenAI-related elements were present in selected components of the broader digital environment, particularly at Minghe Ancient Town, rather than constituting a standardized exposure across both sites. Because visitors encountered these technologies within the overall experience and exposure varied across activities, the empirical measures focus on perceived immersive scene quality at the scene level. This design reflects the naturally occurring experience conditions of the two cases.

Taken together, the two sites represent complementary immersive formats: a concentrated indoor digital-entertainment environment and a distributed heritage-based scenic-area experience. Their technological configurations and spatial forms are not identical, but both organize visitor experience through digitally mediated sensory, narrative, interactive, realism, and usability features. This common experiential structure provides the basis for pooled analysis, while site-specific contextual heterogeneity is acknowledged in the interpretation of the results.

### Participants, procedure, and ethics

Data were collected between June and August 2025, after ethical approval had been obtained, using the same standardized on-site intercept procedure at both study sites. Consistent with on-site visitor research that uses location-based intercepts to capture immediate post-experience evaluations (Baldi et al., 2025), trained researchers approached eligible adult visitors in public buffer areas such as exits, rest zones, waiting areas, and relatively uncrowded corridors, without disrupting normal visitation flows. The same eligibility criteria, consent procedure, questionnaire, and researcher instructions were used at M511 and Minghe Ancient Town. After a brief explanation of the study purpose, estimated completion time, and anonymity assurance, visitors were invited to complete the questionnaire voluntarily. Before responding, all participants read and confirmed an informed-consent statement clarifying that the study was conducted solely for academic analysis and that they could withdraw at any time without adverse consequences. To reduce social desirability bias and situational pressure, respondents were informed that there were no “right answers” and were asked to report their genuine experience. Researchers provided only procedural assistance and did not offer leading explanations of item meanings or value-laden guidance.

Regarding research ethics and data governance, the study involved human participants and was performed in accordance with relevant guidelines and regulations, including the Declaration of Helsinki. Ethical approval was obtained from the Ethics Committee of the Institute of Art and Technology Design, School of Design, NingboTech University (Approval No. NBT-ATD-EC-20250522). Informed consent was obtained from all participants prior to participation (all respondents were adults aged 18 or above). The questionnaire did not collect identifiable information such as names, contact details, facial images, or precise geolocation. All data were recorded anonymously and were accessible only to the research team for statistical analysis and academic purposes. The study did not involve sensitive personal information, medical or psychological interventions, or deceptive procedures; therefore, potential risks were kept at a low level. Findings are reported in aggregated form to prevent individual identification.

### Scale design

All measurement items were derived from established scales or closely related empirical measures in immersive experience, environmental psychology, and sustainable tourism, and were modified to fit the immersive-tourism and ecological-resilience context. All final items were adapted from prior measures, with contextual wording modifications; no item was retained unchanged or newly developed. MIQ drew on multisensory immersion research (Melo et al., 2022; Gougeh and Falk, 2022); NGC on immersive narrative and meaning construction (Taborda-Hernández et al., 2022; Meng and Dolah, 2025); IPA on interactive affordance and participation in virtual tourism (Nguyen et al., 2023; Lee et al., 2025); PRC on realism and authenticity in immersive settings (Choi and Nam, 2024; Shi and Yang, 2026); SU on virtual-reality usability and system-quality research (Othman et al., 2022; Pratisto et al., 2023); NR on nature-relatedness studies (Redondo et al., 2022; Spangenberger et al., 2022); EC on Environmental Concern in immersive and pro-environmental contexts (Meijers et al., 2023; Ibrahim et al., 2025; Kikko and Ishigaki, 2025); EP on the ecological-presence concept (Su et al., 2024); EA on nature-related and virtual-reality awe research (Xu and Hu, 2024; Chirico et al., 2024); EPO on psychological-ownership research (Peck et al., 2021; Wang et al., 2023; Xu et al., 2023); and ESE on Environmental Self-Efficacy research (Chen et al., 2025; Sungur et al., 2025; Plohl et al., 2026). ERS was synthesized and contextually adapted from related measures of environmental policy support, visitor management, and adaptation support (van Valkengoed et al., 2022; Jodłowski et al., 2023; Cao et al., 2024), rather than directly adopted as an existing single scale. All items used a seven-point Likert scale (1 = strongly disagree, 7 = strongly agree).

The initial adapted instrument contained 48 indicators, with four indicators specified for each of the 12 constructs. During measurement-model assessment, indicators were reviewed iteratively by considering relative outer loadings, conceptual redundancy, construct coverage, and the resulting changes in AVE and composite reliability. Item removal was not based on AVE alone. After each deletion, the model was re-estimated, and an indicator was removed only when its exclusion improved or maintained convergent performance without materially weakening the construct’s conceptual coverage. In total, 15 indicators were removed: one from each of nine constructs and two from each of PRC, SU, and ESE. The final specification therefore contained 33 indicators. For reporting clarity, retained indicators were renumbered sequentially within each construct in Table 1.

**TABLE 1:** Measurement scale.

Initial dimension	Code	Item description
Multisensory Integration Quality, MIQ	MIQ1	During this immersive experience, multisensory cues such as visuals and audio were rich and well-coordinated.
	MIQ2	The information from different senses matched consistently and did not make me feel fragmented or conflicted.
	MIQ3	The multisensory presentation enhanced the clarity and intensity with which I perceived natural elements and ecological information.
	NGC1	The sequence of information presentation was reasonable and helped me gradually understand the ecological theme expressed by the scene.
	NGC2	The guidance prompts during the experience were clear and effectively helped me reach key points and obtain information.
	NGC3	The narrative progressed smoothly, and important information was not difficult to understand due to jumps or fragmentation.
Interactive Participatory Affordance, IPA	IPA1	I could easily find entry points for interactive participation (e.g., buttons, tasks, options).
	IPA2	The experience provided multiple ways for me to participate (e.g., exploration, choices, feedback).
	IPA3	The threshold for interactive participation was not high; even without prior experience, I could participate smoothly and move the experience forward.
	PRC1	The scene presentation aligned with my common sense and expectations about real natural environments.
	PRC2	I felt the experience presented an ecologically grounded situation with real-world relevance, rather than merely visual effects.
	SU1	The interface and functional layout were clear, and navigation and switching were smooth.
	SU2	Overall operation was effortless and did not affect my understanding of or engagement with the content.
Nature Relatedness, NR	NR1	I usually feel that I am closely connected with nature.
	NR2	When I come into contact with nature, I often feel that “nature is part of my life.”
	NR3	I tend to understand the relationship between humans and the environment from nature’s perspective.
Environmental Concern, EC Ecological presence, EP	EC1	I pay serious attention to the risks and impacts that environmental changes may bring.
	EC2	When I think about ecological degradation or resource pressure, I feel worried.
	EC3	I believe environmental issues deserve priority and the allocation of public resources to address them.
	EP1	In this experience, I had a strong feeling of being in a natural environment.
	EP2	I felt surrounded by natural elements, as if I were moving within a real ecological space.
	EP3	I felt that the natural environment and ecological changes were right in front of me, rather than distant or abstract.
Ecological awe, EA	EA1	This experience made me feel shocked and filled with admiration.
	EA2	In response to the grandeur or fragility of nature presented in the scene, I felt a strong sense of reverence.
	EA3	After the experience, I felt inspired or uplifted, experiencing an emotional impact that transcended everyday life.
Environmental Psychological Ownership, EPO	EPO1	I regard the relevant natural environment/ecosystem as something related to me and worthy of my concern.
	EPO2	When it faces damage or risk, I feel it is “my own concern” and requires action.
	EPO3	I am willing to invest time or effort to support its long-term stability and improvement.
Environmental Self-Efficacy, ESE	ESE1	I believe I can take practical actions to support ecological stability and risk response.
	ESE2	Even when facing difficulties, I can find ways to advance related actions.
Environmental Resilience Support, ERS	ERS1	I support implementing policies and measures in the local area/destination aimed at enhancing ecological resilience.
	ERS2	I believe promoting such policies is necessary, even if it requires long-term, sustained investment.
	ERS3	I am willing to support the use of public resources to strengthen ecosystems’ capacity to cope with and recover from shocks.

Table 1a details the construct-level refinement of PRC, SU, and ESE, including the original and deleted indicators, the rationale for item removal, and the conceptual coverage of the retained items. PRC was initially represented by PRC1–PRC4; PRC1 and PRC2 were removed, while original PRC3 and PRC4 were retained and renumbered as PRC1 and PRC2. SU was initially represented by SU1–SU4; SU1 and SU3 were removed, while original SU2 and SU4 were retained and renumbered as SU1 and SU2. ESE was initially represented by ESE1–ESE4; ESE2 and ESE4 were removed, while original ESE1 and ESE3 were retained and renumbered as ESE1 and ESE2. The deletion decisions reflected comparatively weaker loadings and, where applicable, overlapping content within each construct rather than a single cutoff criterion.

**TABLE 1a:** Construct-level refinement and conceptual coverage of the two-item measures.

Construct	Initial item pool/item status	Deleted indicators (initial loading)	Retained indicators (original → final)	Rationale and retained conceptual facets
PRC	PRC1–PRC4; all contextually modified from prior realism/authenticity measures	PRC2 (0.650); PRC1 (0.655)	PRC3 → PRC1; PRC4 → PRC2	Deleted items had comparatively weaker loadings and overlapping content. The retained pair covers ecological plausibility/consistency and real-world relevance/credibility. These are core operational facets, but not exhaustive coverage of the full PRC domain.
SU	SU1–SU4; all contextually modified from prior usability/system-quality measures	SU1 (0.581); SU3 (0.589)	SU2 → SU1; SU4 → SU2	Deleted items had clearly lower loadings. The retained pair covers interface/navigation clarity and effortless, continuous operation. These are central operational facets, but provide relatively narrow construct coverage.
ESE	ESE1–ESE4; all contextually modified from prior environmental self-efficacy measures	ESE4 (0.604); ESE2 (0.637)	ESE1 → ESE1; ESE3 → ESE2	Deleted items were comparatively weaker and partly overlapping with retained content. The retained pair covers perceived ability to take practical environmental action and persistence in overcoming difficulties. These represent core capability facets, but do not exhaust the broader ESE domain.

### Data collection

Primary data were collected through structured on-site questionnaires administered to actual visitors at the two immersive tourism settings. The same field protocol was applied at both sites. Questionnaires were distributed in public buffer zones after visitors had completed, or temporarily paused, their experience, helping the responses reflect recent on-site perceptions. Participants self-administered the questionnaire using paper-based forms or mobile devices depending on field conditions, and each respondent was permitted to participate only once. After excluding responses with substantial missing data, logical inconsistencies, or suspected duplicate submissions, 520 valid questionnaires were retained. Of these, 283 were collected at Hangzhou M511 Light and Shadow Hub (54.4%) and 237 at Minghe Ancient Town (45.6%), resulting in a relatively balanced site distribution rather than a sample dominated by one setting.

The sample profile is presented in Table 2. Because the study was designed to examine a common psychological association structure across two related immersive contexts rather than to estimate site-specific effects, the main model uses the pooled sample. The two settings nonetheless differ in spatial form and technological configuration, so site context is treated as a potential source of heterogeneity when interpreting the findings. The use of an identical intercept protocol and a relatively balanced distribution across the two sites reduces procedural differences attributable to data collection, but it does not eliminate contextual variation. Gender distribution in the pooled sample was relatively balanced (47.7% male, 52.3% female). The sample consisted primarily of students (35.0%) and company employees (30.0%), followed by self-employed individuals (21.9%) and government employees (13.1%). Most respondents held a bachelor’s degree (55.0%), while 30.0% held a master’s degree or above and 15.0% had a high school education or below. A majority were first-time visitors to the specific project or setting (65.0%). Detailed frequency statistics are reported in Table 2.

**TABLE 2:** Frequency analysis results.

Category	Option	Frequency	%
Gender	Male	248	47.7
	Female	272	52.3
Occupation	Student	182	35
	Government employee	68	13.1
	Corporate employee	156	30
	Self-employed	114	21.9
Education level	High school or below	78	15
	Bachelor’s degree	286	55
	Master’s degree or higher	156	30
First-time experience of this program/setting	Yes	338	65
	No	182	35
Immersive experience frequency in the past 12 months	0	94	18.1
	1–2 times	208	40
	3–5 times	156	30
	6+ times	62	11.9
Companion type	Alone	130	25
	With friends	234	45
	With family	156	30
Total		520	100

Before responding, all participants were clearly informed of the study purpose, anonymity principles, data usage scope, and their right to withdraw at any time, and they participated voluntarily after reading the informed-consent statement. The study recruited only adults aged 18 years or older. The questionnaire did not collect personally identifiable information such as names, contact details, precise location data, or images. Data were used solely for academic research, and were de-identified after collection, stored in encrypted form, and access was restricted to the research team to protect participant privacy and data security.

### Analytical strategy: structural associations, predictive importance, necessity, and contextual interpretation

This study used PLS-SEM, ANN, NCA, and supplementary interviews to address four complementary analytical questions. PLS-SEM assessed the measurement model and estimated theory-specified structural and indirect associations. ANN compared the predictive importance of measured antecedents of Environmental Resilience Support (ERS) under a potentially non-linear specification. NCA examined whether any antecedent constituted a practically meaningful necessary condition for high ERS. Supplementary interviews provided contextual interpretation of selected quantitative patterns, particularly the non-significant IPA associations and the roles of narrative coherence, realism, responsibility, and efficacy. The methods therefore contribute different forms of evidence: structural association, predictive prioritization, necessity, and contextual interpretation.

Taken together, the analytical sequence distinguishes four questions: which theory-specified associations are statistically supported, which antecedents have greater predictive relevance when considered jointly, whether any antecedent is non-compensable for high ERS, and how selected field patterns can be understood in context. This division of labor allows each method to address a specific inferential purpose without treating similarity across outputs as methodological triangulation.

In Stage 1, PLS-SEM was used to assess the measurement model and estimate the theoretically specified structural and indirect associations. Because the data are cross-sectional, these estimates are interpreted as theory-consistent statistical relationships. In Stage 2, a feedforward multilayer perceptron ANN was applied with ERS as the outcome variable to compare model-specific predictive importance and explore potential non-linear associations. Variables from different levels of the proposed model were entered simultaneously, so ANN importance values are interpreted as predictive rankings. In Stage 3, NCA was used to determine whether any measured antecedent represented a practically meaningful necessary condition for high ERS. Statistical significance was considered together with effect size and ceiling-zone magnitude, allowing necessity to be distinguished from structural association and predictive importance.

Supplementary semi-structured interviews were conducted after the quantitative analysis to clarify selected findings. The interpretation focused on three issues: the consistent roles of narrative coherence and perceived realism, the non-significant IPA pattern, and visitors’ understandings of responsibility and action capability. The interviews thus add contextual detail to quantitative patterns that coefficients and importance rankings alone cannot fully explain.

## Results

### Data analysis and hypothesis testing

Hypotheses were tested using Partial Least Squares Structural Equation Modeling (PLS-SEM) in SmartPLS 3.0 (Cheah et al., 2020).

### Measurement model assessment

#### Reliability and convergent validity

The reflective measurement model was assessed using outer loadings, Cronbach’s alpha, rho_A, composite reliability (rho_c), and average variance extracted (AVE). In the initial 48-item specification, AVE values ranged from 0.417 to 0.487 and composite reliability values from 0.538 to 0.659. Following the construct-level refinement procedure described above, the final specification retained 33 indicators. Outer loadings in the refined model ranged from 0.681 to 0.813. Most exceeded 0.70, while a small number were retained despite slightly lower loadings because they represented distinct aspects of their constructs.

In the refined measurement model, AVE values ranged from 0.500 to 0.618 and composite reliability from 0.750 to 0.791. EPO and ESE, which are central to the proposed responsibility–efficacy framework, had AVE values of 0.529 and 0.603, respectively. Cronbach’s alpha and rho_A were relatively modest for several constructs and were particularly low for the two-item PRC, SU, and ESE measures (alpha/rho_A = 0.371/0.375, 0.383/0.384, and 0.341/0.343, respectively). Their final indicator loadings were 0.752–0.813, 0.772–0.800, and 0.756–0.796, respectively, with AVE values above 0.60 and composite reliability values above 0.75. These results indicate acceptable variance extraction and composite reliability, but the low alpha and rho_A values point to limited internal consistency and relatively narrow construct coverage for the three two-item measures.

Table 3 summarizes the measurement properties of the twelve constructs, while Table 1a details the refinement and conceptual coverage of PRC, SU, and ESE. The retained PRC items capture ecological plausibility and real-world relevance; the SU items capture navigation/interface clarity and operational continuity; and the ESE items capture perceived action capability and persistence in overcoming difficulties. These indicators represent core operational facets of the constructs, although they do not exhaust their broader conceptual domains.

**TABLE 3:** Measurement model assessment.

Construct	Indicators (loading)	No. of items	AVE	Cronbach’s α	rho_A	rho_c
MIQ	MIQ1 (0.725); MIQ2 (0.727); MIQ3 (0.781)	3	0.555	0.601	0.607	0.789
NGC	NGC1 (0.723); NGC2 (0.682); NGC3 (0.734)	3	0.509	0.522	0.523	0.756
IPA	IPA1 (0.707); IPA2 (0.781); IPA3 (0.681)	3	0.524	0.549	0.559	0.767
PRC	PRC1 (0.752); PRC2 (0.813)	2	0.613	0.371	0.375	0.760
SU	SU1 (0.772); SU2 (0.800)	2	0.618	0.383	0.384	0.764
NR	NR1 (0.740); NR2 (0.681); NR3 (0.722)	3	0.511	0.521	0.523	0.758
EC	EC1 (0.711); EC2 (0.776); EC3 (0.694)	3	0.530	0.566	0.576	0.771
EP	EP1 (0.682); EP2 (0.708); EP3 (0.730)	3	0.500	0.501	0.502	0.750
EA	EA1 (0.758); EA2 (0.729); EA3 (0.753)	3	0.558	0.603	0.604	0.791
EPO	EPO1 (0.700); EPO2 (0.743); EPO3 (0.739)	3	0.529	0.556	0.558	0.771
ESE	ESE1 (0.756); ESE2 (0.796)	2	0.603	0.341	0.343	0.752
ERS	ERS1 (0.758); ERS2 (0.733); ERS3 (0.716)	3	0.541	0.576	0.576	0.780

Accordingly, findings involving PRC, SU, and ESE, particularly those involving ESE, are interpreted with caution in the subsequent structural and predictive analyses.

### Discriminant validity

Discriminant validity was examined using the Fornell–Larcker criterion and HTMT (Tables 4, 5). The square root of the AVE for each construct exceeded its correlations with the other constructs, and all HTMT point estimates were below the conventional threshold of 0.90. The highest HTMT value was 0.889 for PRC–ERS, followed by 0.869 for EPO–ESE. Although these values remain below the threshold, their proximity to 0.90 suggests relatively limited empirical separation and warrants cautious interpretation. Conceptually, PRC captures the credibility and real-world relevance of scene presentation, whereas ERS reflects support for resilience-oriented governance; EPO concerns environmental self-relevance and responsibility, whereas ESE reflects perceived action capability. Overall, the discriminant-validity results support the distinction among the constructs, while the relatively high PRC–ERS and EPO–ESE values remain an important measurement qualification.

**TABLE 4:** Fornell–Larcker criterion.

Construct	EA	EC	EP	EPO	ERS	ESE	IPA	MIQ	NGC	NR	PRC	SU
EA	0.747	–	–	–	–	–	–	–	–	–	–	–
EC	0.346	0.728	–	–	–	–	–	–	–	–	–	–
EP	0.392	0.298	0.707	–	–	–	–	–	–	–	–	–
EPO	0.411	0.278	0.281	0.728	–	–	–	–	–	–	–	–
ERS	0.465	0.348	0.386	0.395	0.736	–	–	–	–	–	–	–
ESE	0.279	0.298	0.293	0.378	0.354	0.776	–	–	–	–	–	–
IPA	0.221	0.146	0.258	0.195	0.311	0.217	0.724	–	–	–	–	–
MIQ	0.248	0.169	0.260	0.251	0.358	0.196	0.190	0.745	–	–	–	–
NGC	0.324	0.173	0.344	0.199	0.439	0.202	0.325	0.204	0.713	–	–	–
NR	0.419	0.338	0.334	0.326	0.408	0.300	0.228	0.205	0.281	0.715	–	–
PRC	0.361	0.249	0.282	0.223	0.409	0.222	0.280	0.262	0.331	0.306	0.783	–
SU	0.286	0.207	0.294	0.194	0.385	0.207	0.295	0.234	0.245	0.242	0.207	0.786

**TABLE 5:** HTMT results.

Construct	EC	EA	EP	EPO	ERS	ESE	IPA	MIQ	NGC	NR	PRC
EA	–	–	–	–	–	–	–	–	–	–	–
EP	0.550	0.715	–	–	–	–	–	–	–	–	–
EPO	0.477	0.709	0.529	–	–	–	–	–	–	–	–
ERS	0.601	0.791	0.717	0.696	–	–	–	–	–	–	–
ESE	0.676	0.615	0.706	0.869	0.797	–	–	–	–	–	–
IPA	0.269	0.389	0.478	0.349	0.555	0.499	–	–	–	–	–
MIQ	0.306	0.409	0.470	0.435	0.607	0.433	0.330	–	–	–	–
NGC	0.321	0.575	0.662	0.363	0.795	0.489	0.594	0.357	–	–	–
NR	0.604	0.747	0.649	0.600	0.742	0.713	0.423	0.363	0.532	–	–
PRC	0.521	0.763	0.651	0.481	0.889	0.624	0.620	0.541	0.753	0.690	–
SU	0.427	0.595	0.669	0.421	0.820	0.571	0.652	0.489	0.549	0.542	0.550

### Common method bias

Procedural and statistical measures were used to reduce and assess common method bias. Procedurally, participation was anonymous and voluntary, respondents were informed that there were no correct or socially desirable answers, and researchers avoided providing evaluative guidance during questionnaire completion. Statistically, Harman’s single-factor test indicated that the first unrotated factor accounted for 17.47% of the total variance, below the conventional 50% benchmark. Although this diagnostic cannot completely rule out common method bias, together with the procedural safeguards, it provides limited evidence that a dominant single-method factor was unlikely to account for the observed relationships (Podsakoff et al., 2003).

### Structural model evaluation

This study employed SmartPLS to conduct structural equation modeling; the model specification and results are presented in Figure 2.

**FIGURE 2:**
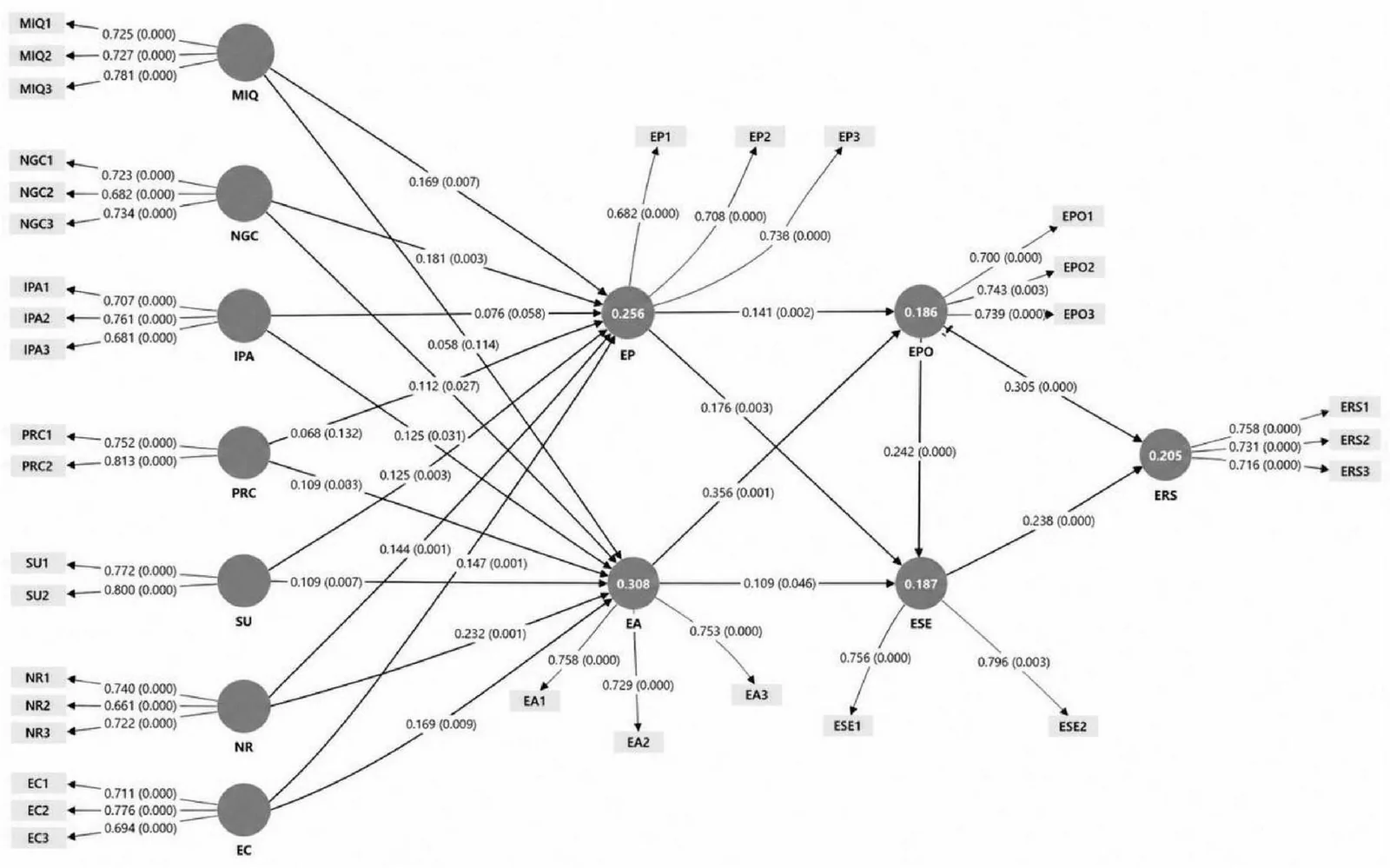
Structural equation modeling analysis results.

### Model fit evaluation

The structural model explained 30.8% of the variance in EA, 25.6% in EP, 18.6% in EPO, 20.5% in ERS, and 18.7% in ESE. Adjusted R^2^ values were 0.299, 0.246, 0.183, 0.202, and 0.182. The SRMR was 0.067 for the saturated model and 0.092 for the estimated model. The fit evidence is mixed. Interpretation therefore focuses on the path estimates and their uncertainty rather than treating global fit as uniformly strong.

### Path analysis

This study used SmartPLS to estimate the proposed structural associations and test the hypotheses. The results are as follows (Table 6).

**TABLE 6:** Path analysis.

Path	β	SE	t	*P*
EA -> EPO	0.356	0.040	8.884	<0.001
EA -> ESE	0.090	0.045	1.978	0.048
EC -> EA	0.169	0.043	3.937	<0.001
EC -> EP	0.147	0.045	3.255	0.001
EP -> EPO	0.141	0.045	3.165	0.002
EP -> ESE	0.176	0.042	4.150	<0.001
EPO -> ERS	0.305	0.040	7.617	<0.001
EPO -> ESE	0.292	0.044	6.682	<0.001
ESE -> ERS	0.238	0.043	5.579	<0.001
IPA -> EA	0.009	0.043	0.213	0.831
IPA -> EP	0.068	0.043	1.579	0.114
MIQ -> EA	0.076	0.040	1.898	0.058
MIQ -> EP	0.109	0.040	2.700	0.007
NGC -> EA	0.132	0.043	3.039	0.002
NGC -> EP	0.181	0.042	4.308	<0.001
NR -> EA	0.232	0.041	5.603	<0.001
NR -> EP	0.144	0.044	3.281	0.001
PRC -> EA	0.160	0.042	3.789	<0.001
PRC -> EP	0.068	0.045	1.507	0.132
SU -> EA	0.109	0.040	2.696	0.007
SU -> EP	0.125	0.042	2.984	0.003

The structural results show a differentiated pattern. MIQ, NGC, and SU were positively associated with EP, whereas PRC and IPA were not. NR and EC were also positively associated with EP.

For EA, NGC, PRC, and SU were positively associated with the outcome. MIQ also showed a positive coefficient (β = 0.076, *p* = 0.058), although it did not reach the conventional 0.05 significance threshold. This pattern suggests a weaker, less conclusive association with EA than with EP. IPA remained non-significant. NR and EC were positively associated with EA. Overall, the five scene-quality dimensions showed differentiated relationships across the two psychological gateways.

At the downstream stage, EP was positively associated with EPO and ESE. EA showed a strong positive association with EPO and a smaller association with ESE (β = 0.090, *p* = 0.048). EPO was positively associated with ESE and ERS, and ESE was positively associated with ERS. Together, these relationships are consistent with the proposed responsibility–efficacy ordering.

The structural results show a selective pattern across the scene-quality dimensions. The central responsibility–efficacy relationships were positive and significant, while several upstream scene-quality paths were not supported.

### Indirect association analysis

Indirect associations were examined for the theoretically specified paths. Table 7 reports the significant indirect associations that reach ERS.

**TABLE 7:** Key significant indirect associations reaching Environmental Resilience Support.

Indirect path	β	SE	t	*P*
EA -> EPO -> ERS	0.109	0.021	5.183	< .001
EA -> EPO -> ESE -> ERS	0.025	0.006	4.036	< .001
EA -> EPO -> ERS	0.043	0.016	2.757	0.006
EP -> ESE -> ERS	0.042	0.014	3.015	0.003
EP -> EPO -> ESE -> ERS	0.010	0.004	2.587	0.010
EPO -> ESE -> ERS	0.070	0.015	4.578	< .001
NR -> EA -> EPO -> ERS	0.025	0.007	3.730	< .001
NR -> EA -> EPO -> ESE -> ERS	0.006	0.002	3.098	0.002
EC -> EA -> EPO -> ERS	0.018	0.006	2.976	0.003
EC -> EA -> EPO -> ESE -> ERS	0.004	0.001	2.803	0.005
NGC -> EA -> EPO -> ERS	0.014	0.006	2.485	0.013
NGC -> EA -> EPO -> ESE -> ERS	0.003	0.001	2.349	0.019
NGC -> EP -> ESE -> ERS	0.008	0.003	2.520	0.012
NGC -> EP -> EPO -> ERS	0.008	0.003	2.262	0.024
PRC -> EA -> EPO -> ERS	0.017	0.006	3.052	0.002
PRC -> EA -> EPO -> ESE -> ERS	0.004	0.001	2.791	0.005
SU -> EA -> EPO -> ERS	0.012	0.005	2.276	0.023
SU -> EA -> EPO -> ESE -> ERS	0.003	0.001	2.206	0.027
SU -> EP -> ESE -> ERS	0.005	0.003	2.068	0.039

Ecological awe retained a significant indirect association with ERS through EPO and through the ordered EA → EPO → ESE → ERS path. The simpler EA → ESE → ERS indirect association was not significant (β = 0.021, *p* = 0.085). EP showed significant indirect associations through EPO, through ESE, and through EP → EPO → ESE → ERS. The EPO → ESE → ERS indirect association also remained significant (β = 0.070, *p* < 0.001). These patterns are consistent with the proposed responsibility–efficacy structure.

Nature Relatedness and EC retained several significant indirect associations with ERS, especially through EA and EPO. This suggests that pre-existing ecological orientations remain relevant to the responsibility-related route.

Among the scene-quality dimensions, NGC showed significant indirect associations through both EA- and EP-based routes. PRC and SU showed significant indirect associations mainly through EA and EPO. MIQ did not retain a significant indirect association reaching ERS in the focused table, and IPA remained unsupported.

### ANN results

Following the PLS-SEM analysis, ANN was used as a prediction-oriented complement to compare the relative importance of the measured antecedents of ERS under a potentially non-linear specification (Ferasso and Alnoor, 2022). Its role is to prioritize predictors within the observed data structure rather than to reproduce the theory-specified path model.

The ANN analysis employed a feedforward multilayer perceptron trained using the backpropagation algorithm. As shown in Figure 3, the input layer included 11 latent-variable scores corresponding to MIQ, NGC, IPA, PRC, SU, NR, EC, EP, EA, EPO, and ESE, and the output layer represented ERS. All inputs were standardized before training, and 10-fold cross-validation was used to evaluate predictive stability and reduce the risk of overfitting. Because variables from different levels of the proposed model were entered simultaneously, the resulting importance values are interpreted as model-specific predictive rankings.

**FIGURE 3:**
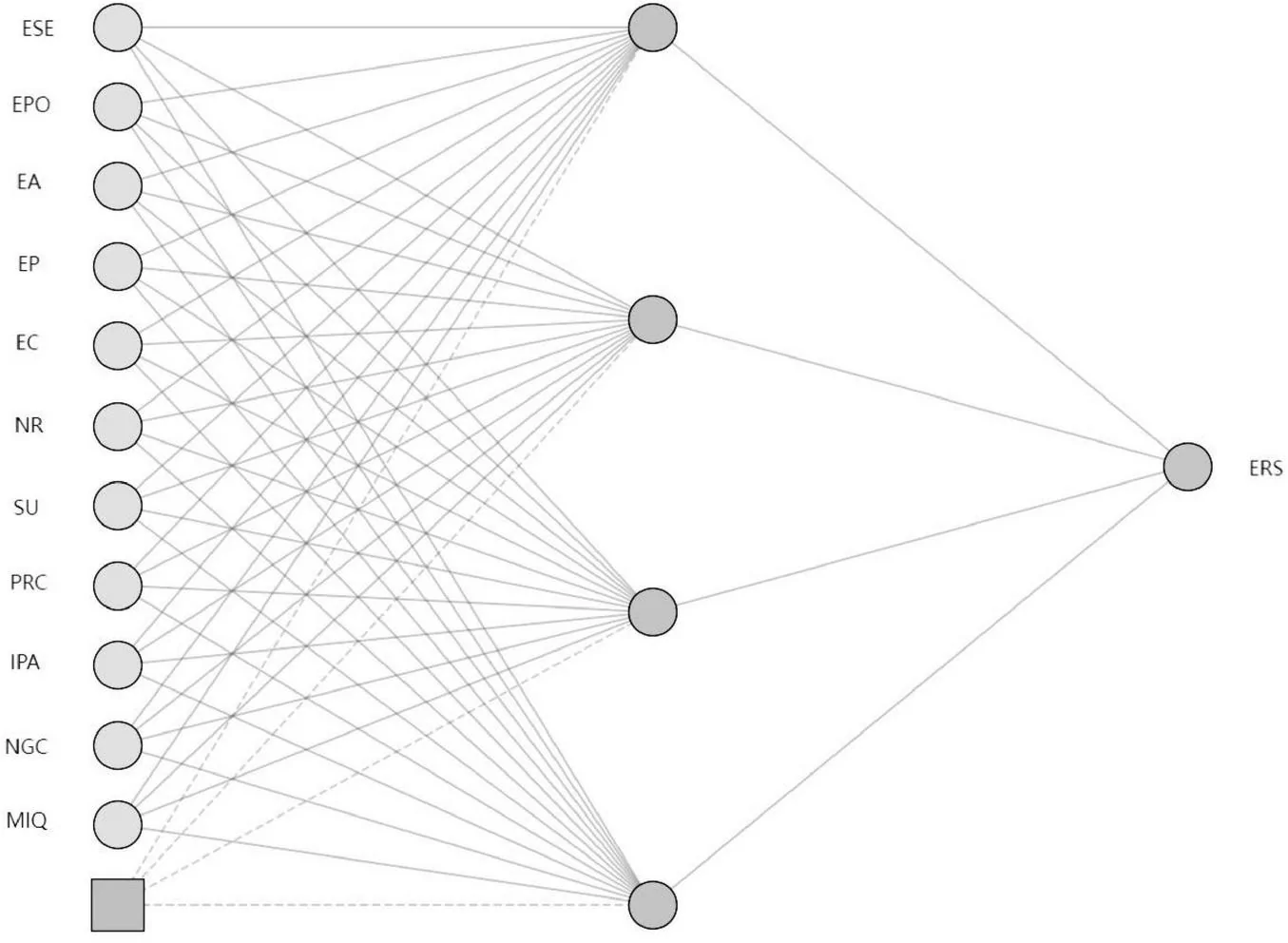
ANN model.

A sensitivity analysis was conducted to compare the relative predictive contribution of each input to ERS. These importance scores answer a different question from PLS-SEM path coefficients: they indicate which measured variables contribute most strongly to ERS prediction when all inputs are considered jointly under the specified non-linear model.

Across the 10 cross-validation folds, training RMSE ranged from 0.1030 to 0.1180 (mean = 0.1119), while testing RMSE ranged from 0.1000 to 0.1183 (mean = 0.1118). The similarity between training and testing errors indicates stable predictive performance within the present sample. External validation across additional settings and populations remains an important next step.

The sensitivity analysis reported in Table 8 ranks the relative predictive importance of the 11 inputs for ERS. Environmental Self-Efficacy (ESE) showed the highest normalized importance (83.05%), followed by Ecological Awe (EA; 79.99%), Ecological Presence (EP; 74.60%), and Environmental Psychological Ownership (EPO; 73.82%). Nature Relatedness (NR; 71.98%), and Environmental Concern (EC; 71.73%) occupied intermediate positions, while the five scene-quality dimensions showed comparatively lower importance: NGC (66.10%), PRC (65.78%), SU (64.39%), MIQ (63.50%), and IPA (61.80%). The ranking provides a prediction-oriented prioritization of the measured antecedents within this ANN specification. Given the comparatively weak internal-consistency evidence for the two-item ESE measure, its high ANN ranking should be interpreted cautiously within the present sample.

**TABLE 8:** Predictive accuracy of the ANN model.

Neural network	Training		Testing	
	*N*	RMSE	*N*	RMSE
ANN1	373	0.1112	147	0.1145
ANN2	368	0.1053	152	0.1083
ANN3	367	0.1126	153	0.1182
ANN4	371	0.1154	149	0.117
ANN5	370	0.103	150	0.1
ANN6	369	0.1178	151	0.1183
ANN7	369	0.115	151	0.1066
ANN8	369	0.1097	151	0.1135
ANN9	366	0.1112	154	0.1179
ANN10	374	0.118	146	0.1036
Mena		0.11192		0.11179
SD	0.00		0.01	

Overall, the ANN results indicate a layered predictive pattern in which proximal responsibility- and capability-related constructs rank above value orientations and scene-quality inputs. The result complements the structural analysis by showing how the measured variables are prioritized when considered jointly in a non-linear predictive model (Table 9).

**TABLE 9:** Sensitivity analysis.

Neural network	ESE	EA	EP	EPO	NR	EC	NGC	PRC	SU	MIQ	IPA
ANN1	0.2319	0.2038	0.3167	0.2697	0.3251	0.2335	0.2776	0.2194	0.3296	0.2911	0.2601
ANN2	0.3466	0.3196	0.3335	0.3042	0.2559	0.2491	0.2586	0.2752	0.2878	0.2216	0.2270
ANN3	0.2933	0.2552	0.2816	0.3000	0.2124	0.2729	0.2197	0.27	0.2845	0.3323	0.3226
ANN4	0.2933	0.2920	0.2348	0.3320	0.2201	0.2449	0.3398	0.2728	0.3192	0.2261	0.2413
ANN5	0.3098	0.2643	0.2339	0.3424	0.2310	0.2720	0.2283	0.3334	0.2909	0.2619	0.2832
ANN6	0.3197	0.2210	0.2776	0.2709	0.2202	0.3370	0.2300	0.2795	0.2121	0.3106	0.24
ANN7	0.2366	0.2514	0.2233	0.3201	0.2921	0.2889	0.2084	0.3309	0.2361	0.3201	0.2541
ANN8	0.2388	0.3341	0.2250	0.3459	0.2546	0.2189	0.2186	0.3087	0.2581	0.3212	0.2006
ANN9	0.3223	0.2317	0.2414	0.2166	0.2960	0.2096	0.2248	0.323	0.2367	0.2571	0.2253
ANN10	0.3083	0.3434	0.2968	0.2424	0.3237	0.3018	0.2617	0.2818	0.3214	0.2233	0.341
Mean relative importance	0.29006	0.27165	0.26646	0.29442	0.26311	0.26286	0.24675	0.28947	0.27764	0.27653	0.25952
Normalized relative importance	83.05%	79.99%	74.60%	73.82%	71.98%	71.73%	66.10%	65.78%	64.39%	63.50%	61.80%

Table 10 compares ANN predictive importance with PLS-SEM total associations with ERS. Because several ANN inputs relate to ERS indirectly in the structural model, total associations provide the most appropriate descriptive reference. Both approaches place ESE, EA, EP, and EPO among the highest-ranked variables, followed by NR and EC, while the five scene-quality dimensions occupy lower positions. The comparison is used to examine broad prioritization patterns across two analytically distinct approaches.

**TABLE 10:** Comparison of Partial Least Squares Structural Equation Modeling (PLS-SEM) total effects and ANN predictive importance.

Predictor	PLS-SEM total effect	ANN importance (%)	PLS-SEM rank	ANN rank
ESE	0.2467	83.05%	1	1
EA	0.1905	79.99%	2	2
EP	0.1065	74.60%	3	3
EPO	0.0762	73.82%	4	4
NR	0.0597	71.98%	5	5
EC	0.0511	71.73%	6	6
NGC	0.0447	66.10%	7	7
PRC	0.0414	65.78%	8	8
SU	0.0291	64.39%	9	9
MIQ	0.0271	63.50%	10	10
IPA	0.0123	61.80%	11	11

The broadly similar prioritization of proximal psychological constructs across PLS-SEM and ANN is useful descriptively. PLS-SEM estimates theory-specified structural and indirect associations, whereas ANN provides a model-specific ranking of predictive importance using the same cross-sectional sample and construct scores. Their comparison therefore highlights convergence in prioritization while preserving the different analytical purposes of the two methods.

### NCA results

Table 11 reports the NCA results using the ceiling envelopment (CE) and ceiling regression (CR) approaches. Although several CR-based p-values were below 0.05, including those for EC, EA, EP, and NGC, all necessity effect sizes were substantively negligible (d = 0.001–0.004), with ceiling zones close to zero (0.001–0.003). None of the examined antecedents therefore constituted a practically meaningful necessary condition for high ERS. This finding adds an important distinction to the structural and predictive results: variables can be strongly associated with, or predictive of, ERS without functioning as non-compensable bottlenecks.

**TABLE 11:** NCA results.

Condition	Method	Accuracy (%)	Ceiling zone	Range	Effect size (d)	*P*-value
EC	CE	100.00%	0.002	0.972	0.002	0.135
	CR	95.97%	0.002	0.993	0.001	0.046
EA	CE	100.00%	0.001	0.986	0.001	0.079
	CR	100.00%	0.002	0.999	0.002	0.031
EP	CE	100.00%	0.003	0.981	0.003	0.168
	CR	96.98%	0.002	0.986	0.001	0.037
EPO	CE	100.00%	0.001	0.991	0.004	0.112
	CR	97.02%	0.001	0.993	0.002	0.187
ESE	CE	100.00%	0.002	0.996	0.002	0.176
	CR	97.01%	0.001	0.994	0.003	0.170
IPA	CE	100.00%	0.001	0.992	0.003	0.149
	CR	98.02%	0.002	0.982	0.001	0.053
MIQ	CE	100.00%	0.002	0.988	0.002	0.124
	CR	97.00%	0.001	0.990	0.002	0.067
NGC	CE	100.00%	0.001	0.983	0.003	0.158
	CR	96.99%	0.002	0.987	0.001	0.042
NR	CE	100.00%	0.002	0.994	0.002	0.103
	CR	97.01%	0.001	0.992	0.003	0.181
PRC	CE	100.00%	0.003	0.989	0.004	0.139
	CR	98.00%	0.002	0.995	0.002	0.059

### Bottleneck analysis

For ERS targets from 0% to 90%, the bottleneck analysis identified no meaningful minimum thresholds. At the 100% target, a few low bottleneck values appeared, but the associated necessity effect sizes remained trivial. The NCA results therefore suggest that high ERS is not tied to a universal minimum level of any single scene-quality, value, responsibility, or efficacy variable.

### Supplementary interview explanation

#### Purpose

The supplementary interviews focused on selected quantitative findings: the roles of narrative coherence and perceived realism in EP and EA, the non-significant role of IPA, and visitors’ understandings of responsibility and action capability.

#### Methods

Follow-up semi-structured interviews were conducted with six participants drawn from the same immersive tourism settings after the survey was completed. Each interview lasted approximately 20–30 min and explored how participants interpreted scene organization, ecological presence and awe, responsibility, and action capability. Given the small supplementary sample, the interview material is used for contextual interpretation rather than for broad qualitative generalization.

The interview protocol covered three areas: (1) perceptions of narrative, realism, interaction, and system usability; (2) interpretations of ecological presence, ecological awe, and the IPA pattern; and (3) understandings of environmental responsibility and self-efficacy in relation to resilience support. All interviews were conducted with informed consent and were transcribed and organized for interpretive analysis.

#### Supplementary interview insights

First, participants generally reported stronger ecological involvement when narrative guidance, realism cues, and system usability worked coherently. Coherent narratives and credible ecological cues helped them understand environmental change and remain engaged with the ecological situation. These accounts provide contextual insight into the observed NGC and PRC patterns.

Second, participants’ accounts help explain the non-significant IPA results. Interaction was not always experienced as ecologically meaningful; task completion, repeated choices, and interface demands sometimes redirected attention from ecological content toward operation. Interaction was viewed as more useful when it clarified ecological causality, supported role-taking, or made environmental consequences understandable. This pattern suggests that the value of interaction depends on how closely participation is integrated with ecological meaning.

Third, participants distinguished between feeling situated in an ecological setting and experiencing an emotionally elevated response, while also describing environmental issues as becoming more personally relevant and actionable after the experience. These accounts illustrate the practical meanings of EP, EA, EPO, and ESE and provide context for interpreting the quantitative relationships.

## Discussion

The results support two related but distinct psychological gateways. EP is associated with multisensory integration, narrative coherence, system usability, and ecological orientations, consistent with research linking sensory coordination and coherent immersive environments to presence and sustained attention (Melo et al., 2022; Su et al., 2024). EA is associated with narrative coherence, perceived realism, system usability, and ecological orientations, in line with work showing that awe is more likely when immersive content makes natural vastness, fragility, and meaning salient (Chirico et al., 2024; Xu and Hu, 2024). Both gateways are also related to responsibility- and capability-related beliefs, while the EA–ESE association is comparatively small. The significant EPO → ESE → ERS indirect association is consistent with the proposed responsibility–efficacy ordering. Because the data are cross-sectional, the parallel EP/EA specification is best understood as a theoretically organized pattern of relationships; longitudinal or experimental work will be needed to establish whether presence and awe unfold sequentially or reciprocally.

Partial Least Squares Structural Equation Modeling, ANN, NCA, and the supplementary interviews provide complementary views of ERS. PLS-SEM estimates the theory-specified association structure; ANN prioritizes predictors under a non-linear specification; NCA evaluates whether any antecedent functions as a necessary condition; and the interviews contextualize selected field patterns. Together, these analyses indicate that responsibility- and capability-related constructs are comparatively important for explaining and predicting ERS, while no single antecedent emerges as an indispensable minimum condition. The additional methods therefore extend the interpretation of the structural model by distinguishing association, prediction, necessity, and contextual explanation.

The scene-quality results also show that the five dimensions do not operate in the same way. NGC and SU are associated with both EP and EA. The role of narrative coherence is consistent with studies showing that structured storytelling helps users follow meaning, maintain immersion, and connect information across an experience (Taborda-Hernández et al., 2022; Chang and Suh, 2025; Meng and Dolah, 2025). The positive role of SU also fits research showing that unstable or difficult systems can interrupt immersion and reduce experiential continuity (Othman et al., 2022; Pratisto et al., 2023). MIQ was significantly associated with EP, whereas its association with EA was positive but did not reach the conventional 0.05 threshold (β = 0.076, *p* = 0.058). This near-threshold pattern suggests that multisensory integration may still have some relevance to ecological awe, but the evidence is not sufficiently strong to support H3a in the present sample. One possible interpretation is that coordinated multisensory cues primarily support situated presence, whereas awe may additionally depend on interpretive factors such as narrative meaning, perceived realism, or the perceived vastness and fragility of ecological content. Future research should re-examine this association using larger samples or experimental manipulations of multisensory intensity and ecological content. By contrast, PRC is associated with EA but not EP. This may indicate that credible ecological cues are especially important when visitors interpret the scene as meaningful and consequential (Choi and Nam, 2024; Shi and Yang, 2026).

Interactive Participatory Affordance (IPA) was not significantly associated with either EP or EA, and most related indirect associations were unsupported. The result points to a more conditional role for interaction. In the interviews, task completion and interface demands sometimes shifted attention away from ecological content, whereas interaction was viewed more positively when it clarified ecological causality or encouraged role-taking. This interpretation is consistent with research showing that interactivity can support agency but can also increase cognitive demands when poorly integrated with the experience (Lehikko et al., 2024; Breves and Stein, 2023), and with work emphasizing meaningful rather than merely frequent interaction (Lee et al., 2025). Future research could examine boundary conditions such as interaction depth, task meaningfulness, feedback immediacy, cognitive load, visitor involvement, digital familiarity, and integration with ecological narratives through moderation, subgroup, or experimental designs.

Nature Relatedness and EC were positively associated with both EP and EA. Visitors therefore did not respond to the same scene in the same way. Those with stronger connections to nature or greater Environmental Concern were more likely to attend to ecological cues and interpret them as personally relevant. This is consistent with studies linking Nature Relatedness to stronger engagement with nature-based cues (Brambilla et al., 2024; Desrochers et al., 2024) and with research showing that Environmental Concern shapes responses to immersive environmental information (Meijers et al., 2023; Ibrahim et al., 2025). The result also fits evidence that Environmental Concern and risk perception influence pro-environmental intentions across contexts (Kikko and Ishigaki, 2025). The interviews suggested a similar pattern. Some participants entered the experience with clear concern about ecological fragility, while others first approached it as a digital attraction. Immersive experience may therefore amplify existing ecological orientations rather than replace them.

Environmental Resilience Support focuses on support for resilience-oriented governance rather than general green intention. EPO showed a stronger direct association with ERS (β = 0.305) than ESE (β = 0.238), and EPO was also positively associated with ESE. This pattern is consistent with research linking psychological ownership to stewardship and pro-environmental action (Peck et al., 2021; Wang et al., 2023; Xu et al., 2023) and with evidence that self-efficacy supports pro-environmental action when individuals believe their contribution can be effective (Sungur et al., 2025; Plohl et al., 2026; Qiu, 2025). The significant EPO → ESE → ERS indirect association is consistent with the proposed responsibility–efficacy structure, although ESE-related findings should be interpreted cautiously in light of the measurement limitations noted above.

### Theoretical implications

First, the study distinguishes ecological presence from ecological awe as two related but non-identical gateways. The results sharpen this distinction because the same scene-quality variables are not associated with both gateways in the same way. NGC and SU are associated with both; MIQ shows a clear association with EP and a weaker, near-threshold association with EA; PRC is associated with EA but not EP. These differentiated patterns support treating situated entry and self-transcendent emotion as distinct dimensions of ecological experience.

Second, the study develops a focused responsibility–efficacy explanation of ERS by connecting the responsibility logic associated with NAM and the capability logic associated with TPB. EPO captures environmental self-relevance and responsibility, whereas ESE captures confidence in effective action. Their positive relationships with ERS suggest that resilience-oriented support is associated with both perceived responsibility and perceived capability, extending these psychological logics into immersive environmental experience research.

Third, the supplementary analyses clarify the position of responsibility and capability within the broader model. PLS-SEM places EPO and ESE in proximal positions within the theory-specified association structure, ANN assigns comparatively high predictive importance to these constructs, and NCA identifies no practically meaningful necessary antecedent. This combination suggests that responsibility and capability are comparatively important correlates of ERS without functioning as universal minimum conditions.

Fourth, the findings qualify the role of immersive scene-quality dimensions. NGC shows the most consistent association across EP and EA, while SU is also related to both. MIQ is significantly associated with EP, and its association with EA is positive but falls just above the conventional significance threshold (*p* = 0.058); PRC, by contrast, is associated with EA but not EP. IPA is not significantly associated with either gateway. These differences indicate that scene-quality dimensions should not be treated as equally effective. In particular, the IPA pattern suggests that interaction may become ecologically meaningful only when it is integrated with content, narrative purpose, and visitor characteristics.

### Managerial implications

The findings offer several implications for the design and operation of immersive ecology- and nature-themed experiences in digitally mediated immersive tourism settings. First, managers may consider strengthening visitors’ perceived capacity to support environmental action by providing clear action options, accessible participation routes, and understandable feedback about the possible consequences of individual and collective behavior. This implication is informed by the positive ESE–ERS association and the high predictive ranking of ESE and should be interpreted cautiously in light of the measurement limitations discussed above.

Second, environmental issues should be framed as personally and collectively relevant rather than as distant ecological problems. Since EPO is associated with both ESE and ERS, narratives may connect ecological conditions with local quality of life, place identity, community memory, public safety, and intergenerational wellbeing. Such framing may help visitors view themselves not only as spectators, but also as potential participants in environmental stewardship.

Third, the five scene-quality dimensions should not be treated as interchangeable. Narrative coherence is the most consistent scene-level correlate of both EP and EA, and system usability is also associated with both gateways. Multisensory integration is more strongly linked to presence, while perceived realism is more closely linked to awe. Interaction appears most useful when it serves narrative purpose and ecological meaning; interactive features should therefore be evaluated by whether they reduce distraction, clarify ecological causality, and support meaningful role-taking.

Fourth, design should combine emotional and situated forms of engagement. Scenes that communicate ecological vastness, fragility, and consequence may support awe, while multisensory consistency, smooth transitions, and low-disruption interfaces may support ecological presence. Visitor differences should also be considered. For those with weaker ecological orientations, experiences may emphasize everyday relevance and visible consequences; for more environmentally concerned visitors, deeper interpretive content and follow-up participation opportunities may be appropriate.

Finally, the NCA results caution against relying on a single “wow-factor” feature. A more robust design strategy is to combine coherent narration, credible ecological situations, situated and emotional engagement, and responsibility- and efficacy-related cues. The absence of a practically meaningful necessary condition suggests that high ERS is unlikely to depend on one universal threshold, making coordinated scene design more appropriate than single-factor optimization.

### Limitations and future research

This study has several limitations. First, data were collected through on-site intercept sampling at two immersive tourism settings in China. Although the same field protocol was applied at both sites and the sample distribution was relatively balanced (M511: *n* = 283; Minghe Ancient Town: *n* = 237), the two settings differ in spatial organization, technological configuration, and cultural-tourism function. The pooled analysis was intended to identify a common association structure across these related contexts rather than to establish site equivalence or estimate site-specific effects. Some contextual heterogeneity may therefore remain, which should be considered when generalizing the findings. Future research could employ larger multisite samples and formally examine measurement invariance and structural differences across settings using procedures such as MICOM and PLS-MGA. The sample also included a relatively high proportion of students (35.0%) and respondents with at least a bachelor’s degree (85.0%). This composition may limit the representativeness of the findings across broader visitor populations, particularly those with different educational backgrounds, occupations, and levels of familiarity with digital tourism environments.

Second, the cross-sectional design captures theoretically specified associations at a single point in time and does not establish temporal ordering. Reverse or reciprocal relationships remain possible; for example, Environmental psychological ownership, self-efficacy, or resilience-support orientations may influence retrospective evaluations of immersive scene quality. Similar uncertainty applies to the relationship between ecological presence and ecological awe. Longitudinal surveys, field experiments, pre–post designs, and delayed follow-up measures could help distinguish parallel, sequential, and reciprocal relationships more clearly.

Third, the responsibility–efficacy framework is informed by selected psychological processes associated with NAM and TPB rather than by complete specifications of either theory. The present model therefore does not include constructs such as awareness of consequences, ascription of responsibility, personal norms, attitudes, subjective norms, or broader perceived behavioral control. Future research could incorporate these components and compare the focused framework examined here with more comprehensive theoretical specifications.

Fourth, GenAI-related functionality was present in selected elements of the broader technological context rather than constituting a common or standardized feature across both study settings. GenAI exposure itself was neither directly measured nor experimentally manipulated, and visitors may have encountered different digital functions during their experience. The findings should therefore be interpreted as associations involving perceived immersive scene quality within digitally mediated immersive settings in which some GenAI-related elements were present, rather than as evidence of an independent effect of GenAI. Future research could compare settings with and without specific GenAI functions under controlled conditions or manipulate functions such as adaptive narration, personalization, conversational responsiveness, and real-time content generation while holding ecological content and visual presentation constant. Such designs would allow the contribution of specific GenAI functions to be distinguished from broader scene-design effects.

Fifth, the measurement structure presents an important limitation. All items were contextually modified from prior scales or related empirical measures, and ERS was synthesized from several related measurement traditions rather than directly adopted as a single existing scale. The initial four-item-per-construct instrument was refined through sample-based measurement-model assessment, leaving PRC, SU, and ESE represented by two indicators. Although the retained pairs preserve core operational facets—ecological plausibility and real-world relevance for PRC, interface clarity and operational continuity for SU, and action capability and persistence for ESE—their low Cronbach’s alpha and rho_A values indicate limited internal consistency and relatively narrow coverage of the broader conceptual domains. This issue is especially relevant to ESE given its central role in the proposed framework and its association with ERS. Findings involving these measures should therefore be interpreted cautiously. Future research should re-expand the item pools, conduct independent pilot testing and scale refinement, and validate the measurement structure in separate samples and tourism settings.

Finally, ERS was measured as self-reported support rather than actual governance behavior. Social desirability and the intention–behavior gap may therefore affect the findings. Future research could incorporate observed compliance, policy-choice experiments, public-resource allocation tasks, contribution decisions, and follow-up measures of actual participation to determine whether stated resilience support translates into sustained behavior.

## Data Availability

The datasets presented in this study can be found in online repositories. The names of the repository/repositories and accession number(s) can be found in the article/supplementary material.
